# Supplementary material for: Patterns of Intron Gain and Loss in Fungi
Source: PLoS Biol. 2004 Nov 30;2(12):e422. doi: 10.1371/journal.pbio.0020422 (PMC532390; doi:10.1371/journal.pbio.0020422)
Supplement: Table S1 — Also available at http://genes.mit.edu/NielsenEtAl/. (4.3 MB ZIP). [file pbio.0020422.st001.zip › NielsenEtAl/html/348.html]

AN8045.1.NCU09014.1.MG01676.1.FG06907.1


```
 CLUSTAL W (1.82) Multiple Sequence Alignments - Introns Inserted


Sequence 1: NCU09014.1	264 aa
Sequence 2: FG06907.1	264 aa
Sequence 3: MG01676.1	266 aa
Sequence 4: AN8045.1	297 aa
Alignment Length: 304 aa
Number Identitical Residues: 172 aa
Alignment Score (without introns) 7859


MG01676.1 	MSTITTTALQSGQP----------------------~PIIPLSFNANQPETIRLYPLSNY
NCU09014.1	MSTITPNALQSSVP----------------------~PTIPLPFNANQPETIRLYPLSNY
FG06907.1 	MSTITPDALQSGQA----------------------~PVIPLSFNGNQPEKVTLYPLSNY
AN8045.1  	MSTTTITAIQSSFPCVLPSDPVPSSSKSSSSKLSGH2PIAPKDFSAKQPETIRLYPLSNY
          	*** *  *:**. ..  .:.. .:::.::::. :.  *  *  *..:***.: *******

MG01676.1 	TFGVKETQPEEDPSVLARLKRLEEHYNAHGMRRTCEGILVCHEHNHPHILMLQIANAFFK
NCU09014.1	TFGVKETQPEEDPSVLARLKRLEEHYNAHGMRRTCEGILVCHEHNHPHILMLQIANAFFK
FG06907.1 	TFGVKETQPEEDPSVIARLKRLEEHYTEHGMRRTCEGILVCHEHNHPHILMLQIANAFFK
AN8045.1  	TFGTKETQPEEDPSVLARLKRLEEHYEKHGMRRTCEGILVCHEHNHPHVLMLQIANAFFK
          	***.***********:**********  ********************:***********

MG01676.1 	L~PGDYLRPEDDEVEGFKARLDERLAPVGRLGEGE-EAGDWQIGDCLAQWWRPNFETFM~
NCU09014.1	L~PGDYLRPEDDEIEGFKQRLDERLAPVGSLGEGE-KAGDWQVGDCLAQWWRPNFETFM~
FG06907.1 	L~PGDYLRPEDDEIQGFKSRLDERLAPVGRLGEGE-EAGDWQVGDCLAQWWRPNFETFM~
AN8045.1  	L2PGDYLLHDDDEIEGFKRRLNERLAPVGSQFSGEGVNEDWEIGDTLAQWWRPNFETFI2
          	* *****  :***::*** **:*******   .**.   **::** ************: 

MG01676.1 	YPFVPAHVTRPKECKKLYFIQLPNSK1VLSVPKNMKLLAVPLFELYDNSARYGPQLSAIP
NCU09014.1	YPFVPAHVTRPKECKKLYFIQLPKSK1VLSVPKNMKLLAVPLFELYDNTARYGPQLSAIP
FG06907.1 	YPFIPAHVTRPKECKKLYFIQLPKQK1VLSVPKNMKLLAVPLFELYDNTARYGPQLSAIP
AN8045.1  	KDQMSLVG------HDMLMCLRVAAE~VLSVPKNMKLLAVPLFELYDNNQRYGPQLSSIP
          	   :.         :.: :      : *********************. *******:**

MG01676.1 	HLLSKYNFEFVDEQGNVVAQTPGSGPGEGDHVPKTKVLAGGDDVDMKQEN--------GA
NCU09014.1	HLLSRYNFEFVDENGEVVAVTPGDG--DG-YVPKTKVLASGDDVDMKTEE--------GN
FG06907.1 	HLLSRYNFEFVDENGNVVAATPGSAAPDG-YVPHVKVLA-GDDTDMKEEN--------GT
AN8045.1  	HLLSRYNFEFVDENDNVVAATPGMGS-PGDQVPKTKVLAGDNNGKAKEEDSGMADYTEGA
          	****:********:.:*** *** ..  *. **:.****..:: . * *::. :. :.* 

MG01676.1 	H-------
NCU09014.1	GP------
FG06907.1 	S-------
AN8045.1  	ETGGQQGQ
          	 .......
```
